# Baseline Psychological Traits Contribute to Lake Louise Acute Mountain Sickness Score at High Altitude

**DOI:** 10.1089/ham.2021.0073

**Published:** 2022-03-28

**Authors:** Benjamin James Talks, Catherine Campbell, Stephanie J. Larcombe, Lucy Marlow, Sarah L. Finnegan, Christopher T. Lewis, Samuel J.E. Lucas, Olivia K. Harrison, Kyle T.S. Pattinson

**Affiliations:** ^1^ Population Health Sciences Institute, Newcastle University, Newcastle Upon Tyne, United Kingdom.; ^2^ Birmingham Medical Research Expeditionary Society, Birmingham, United Kingdom.; ^3^ Medical School, University of Birmingham, Birmingham, United Kingdom.; ^4^ Warwick Medical School, Warwick University, Coventry, United Kingdom.; ^5^ Nuffield Department of Clinical Neurosciences, University of Oxford, Oxford, United Kingdom.; ^6^ Department of Anesthesia, Ysbyty Gwynedd, Bangor, United Kingdom.; ^7^ School of Sport, Exercise and Rehabilitation Sciences, University of Birmingham, Birmingham, United Kingdom.; ^8^ Translational Neuromodeling Unit, Institute for Biomedical Engineering, University of Zurich and ETH Zurich, Zurich, Switzerland.; ^9^ School of Pharmacy, University of Otago, Dunedin, New Zealand.

**Keywords:** acute mountain sickness, altitude, breathlessness, exercise, filter detection task, interoception

## Abstract

Talks, Benjamin James, Catherine Campbell, Stephanie J. Larcombe, Lucy Marlow, Sarah L. Finnegan, Christopher T. Lewis, Samuel J.E. Lucas, Olivia K. Harrison, and Kyle T.S. Pattinson. Baseline psychological traits contribute to Lake Louise Acute Mountain Sickness score at high altitude. *High Alt Med Biol.* 23:69–77, 2022.

**Background:**

Interoception refers to an individual's ability to sense their internal bodily sensations. Acute mountain sickness (AMS) is a common feature of ascent to high altitude that is only partially explained by measures of peripheral physiology. We hypothesized that interoceptive ability may explain the disconnect between measures of physiology and symptom experience in AMS.

**Methods:**

Two groups of 18 participants were recruited to complete a respiratory interoceptive task three times at 2-week intervals. The control group remained in Birmingham (140 m altitude) for all three tests. The altitude group completed test 1 in Birmingham, test 2 the day after arrival at 2,624 m, and test 3 at 2,728 m after an 11-day trek at high altitude (up to 4,800 m).

**Results:**

By measuring changes to metacognitive performance, we showed that acute ascent to altitude neither presented an interoceptive challenge, nor acted as interoceptive training. However, AMS symptom burden throughout the trek was found to relate to sea level measures of anxiety, agoraphobia, and neuroticism.

**Conclusions:**

This suggests that the Lake Louise AMS score is not solely a reflection of physiological changes on ascent to high altitude, despite often being used as such by researchers and commercial trekking companies alike.

## Introduction

Acute Mountain Sickness (AMS) is a common feature of ascent to altitude, affecting ∼25% of individuals ascending to moderate altitudes (2,000–3,000 m) and up to 58% of individuals at 4,500 m (Honigman et al., 1993; Schneider et al., 2002). Ascent rate also plays a key role in AMS prevalence, with rapid ascent profiles on Mount Kilimanjaro (5,895 m) resulting in an AMS incidence as high as 75% (Karinen et al., 2008). Individual susceptibility is another important determinant, with more experienced mountaineers tending to suffer less AMS (Mairer et al., 2010).

Lake Louise AMS Score, a self-reported symptom score, is the most widely used measure of AMS (Roach et al., 2018). It is well recognized that symptoms of Lake Louise AMS score are not fully explained by measures of hypoxic insult, including arterial oxygen saturations, respiratory rate, and heart rate (Chen et al., 2012; Wagner et al., 2012). Direct measures of hypoxia on brain function also fail to explain this disparity, including matched regional oxygen saturations, electroencephalography, cerebral blood flow velocity, and cerebral edema (Mairer et al., 2012; Feddersen et al., 2015). Given the diverse array of subjective symptoms induced by ascent to high altitude (Hall et al., 2014), it may be that discrepancies in symptoms reporting between individuals can be explained by differences in their perceptual sensitivity or behavioral profiles.

Interoception refers to an individual's ability to sense the internal state of their body (Simmons and Land, 1987; Barret and Simmons, 2015). The “Bayesian Brain” hypothesis of perception, including interoception (Barret and Simmons, 2015; Stephan et al., 2016), is a popular neuroscientific theory. In brief, the Bayesian Brain Hypothesis proposes that to interpret numerous noisy sensory stimuli (e.g., vision, pain, nausea), the brain generates an internal model of the world, against which it constantly tests new sensory inputs against.

The second-order process assessing the accuracy of this predictive model is known as metacognition, a term used to describe “cognition about cognition” (Stephan et al., 2016), or “insight” into your own perceptions. By extension, as all symptoms are produced centrally in the brain, they cannot be fully explained by measures of peripheral physiology alone. Differences in interoception may help explain the discrepancies in AMS symptomology between individuals on ascent to high altitude.

Ascent to high altitude is associated with an array of physiological and behavioral stressors, including the disruption of multiple physiological symptoms due to AMS (Hall et al., 2014), hypoxia, and its associated systemic inflammatory response (Eltzschig and Carmeliet, 2011), and fatigue resulting from travel across multiple time zones (Stephan et al., 2016). Such stressors have the potential to impair interceptive performance either independently or in combination. Interestingly, habitual exercise is thought to improve interoceptive performance, with athletes demonstrating better matching between ventilation and perceived breathlessness than sedentary controls (Faull et al., 2016).

Furthermore, functional magnetic resonance imaging of brain networks associated with anticipating breathing stimuli has shown brain activity that reflects subsequent interoceptive perceptions in athletes compared with sedentary controls (Faull et al., 2018), and changes in activity in patients with chronic respiratory disease after a course of pulmonary rehabilitation (exercise and education) on exposure to breathlessness cues (Hergistad et al., 2017). Therefore, this study aimed to test the hypothesis that initial ascent to high altitude would impair interoceptive performance, while daily exercise in the form of an 11-day trek at high altitude would act as interoceptive training and thus improve performance.

Individuals' psychology can also play a significant role in their experience of symptoms—framing their internal model of the world according to the Bayesian Brain Hypothesis. In particular, fatigue and anxiety may be the brain's manifestations of poor perceived self-efficacy and control, presenting as a state of learned helplessness (Stephan et al., 2016). Indeed, 1 study of breathlessness in 100 patients with Chronic Obstructive Pulmonary Disease demonstrated different behavioral profiles and brain activity in the anterior insula (a likely key interoceptive center) between a high and low symptom burden group, in the absence of any differences in spirometry between the two groups (Finnegan et al., 2021).

Therefore, we also hypothesized that self-report questionnaires characterizing individuals' baseline psychological state may correlate with their symptom burden over the expedition.

## Methods

### Study design

**FIG. 1.**
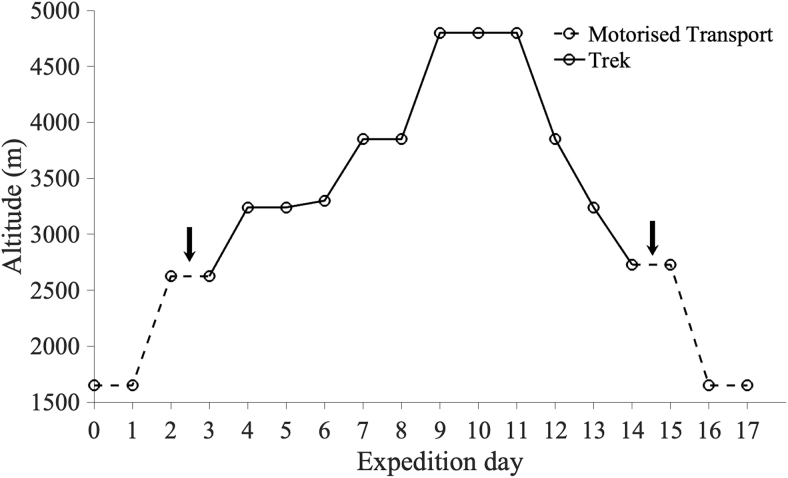
The ascent profile of the altitude group in Sikkim, India. Days of travel by motor vehicle are plotted in a *dashed line* and days of trekking by foot are plotted with a *solid line*. The times of the two interoceptive tests on the expedition are marked by *arrows*.

This study was a two-group repeated measures design, consisting of equally sized altitude and control groups. Both groups completed interoceptive testing at three time points 2 weeks apart. The control group completed all three tests in Birmingham, United Kingdom (140 m). The altitude group completed baseline testing in Birmingham, United Kingdom (140 m); after arrival at high altitude in Lachung, India (2,624 m); and after completing a 11-day trek at altitude in Lachen, India (2,728 m). The ascent profile of the trek is shown on Figure 1, with the highest camp situated at 4,800 m. This study was approved by the Central University Research Ethics Committee of the University of Oxford (Ref: R60699/RE001). All participants provided written consent.

### Study participants

The altitude group was composed of members of the Birmingham Medical Research Expeditionary Society (*n* = 18; 11 males, 7 females; median age ± interquartile range = 30.5 ± 15 years, age range = 23–74 years) taking part in a 2-week trek in Sikkim, India. An age- and sex-matched control group was recruited through advertisement on the University of Birmingham campus (*n* = 18; 11 males, 7 females; median age ± interquartile range = 31 ± 10 years, age range = 23–72 years).

Exclusion criteria for participating in the study included significant medical comorbidities, smoking history, recent travel across multiple time zones, and recent ascent to high altitude (see Supplementary Information S1 for full criteria). Inclusion criteria included being an acceptable age- and sex-match to one of the expedition participants (same sex and ≤8 years age difference).

There was no significant difference in age between the two groups (Wilcoxon Rank-Sum Test, *p* = 0.3353), with a median age difference of 2 years and interquartile range of 3 years between the two groups.

### Primary outcome measures

#### Respiratory interoceptive test

**FIG. 2.**
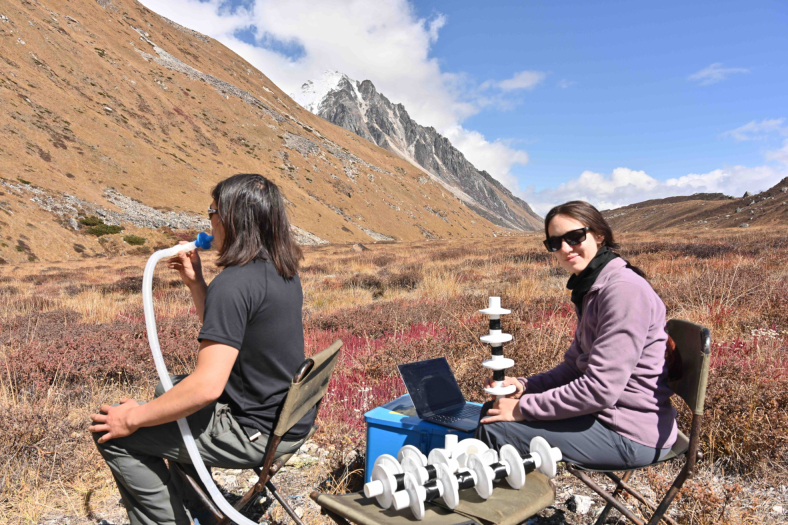
Photograph illustrating the experimental setup of the respiratory interoception (filter detection) task in Sikkim, India. Actual experiments were performed indoors in a hotel room.Credit: Arthur Bradwell. Consent to publish was obtained from subjects in photograph.

A respiratory threshold detection task, the filter detection task (Harrison et al., 2021a), was used as a measure of respiratory interoception. In this task, the participant breathes through a simple breathing system, and following three baseline breaths, either an inspiratory load is added through the addition of clinical breathing filters, or an empty filter (sham) is used (Fig. 2). After each trial, participants are asked to decide whether or not resistance was added, as well as reporting their confidence in their decision on a confidence scale from 0 to 10. The number of filters is varied according to an algorithm that tracks performance, until a threshold is found at which the participant is 60%–85% confident in their response. The task is then repeated at this threshold for 60 trials.

The filter detection task can then be used to determine perceptual sensitivity (number of filters), perceptual bias in symptom reporting (bias toward yes or no), metacognitive bias (average confidence), and metacognitive performance (Mratio, calculated from meta-d′/d)—that is, the ability to accurately reflect upon and thus control cognitive or perceptual processes (Garfinkel et al., 2016a, 2016b).

#### Cardiorespiratory physiology

Basic measures of cardiorespiratory physiology were made noninvasively using pulse oximetry and an automatic sphygmomanometer, measuring oxygen saturations, heart rate, and blood pressure. These measures were collected each morning on the expedition as part of a daily medical review.

#### Self-report scores

Participants were asked to complete a number of self-report scores during each of their three testing sessions, including the Lake Louise AMS Scale (Roach et al., 2018), Multidimensional Assessment of Interoceptive Awareness (Mehling et al., 2012), Fatigue Severity Scale (Krupp et al., 1989), Epworth Sleepiness Scale (Johns, 1991), State-Trait Anxiety Inventory (Spielberger et al., 1983), Center for Epidemiological Studies Depression Scale (Radloff, 1977), Positive and Negative Affect Scale (Watson et al., 1988), Mobility Inventory for Agoraphobia (Chambless et al., 1985), Anxiety Sensitivity Index (Reiss et al., 1986), U.K. Biobank Neuroticism Scale (Smith et al., 2013), and WHO Global Physical Activity Scale (World Health Organization, 2002) reported in metabolic equivalent minutes.

The altitude group also completed daily Lake Louise AMS scores throughout the trek each morning as part of daily medical review.

### Statistical analysis

Statistical analysis was performed according to the prepublished statistical analysis plan (https://osf.io/zgj9c/).

#### Examining interoceptive performance

The respiratory threshold task was analyzed using the hierarchical HMeta-d statistical model (Harrison et al., 2021a); model fits were implemented in MATLAB (Mathworks, Natick) with sampling conducted using JAGS (Plummer, 2003). The HMeta-d model was fitted separately for each pair of tests in the control group and altitude group to look at the effect of initial ascent to high altitude (visit 1 and 2) and of an 11-day trek at altitude (visit 2 and 3).

Mratio was compared between each visit by calculating a one-tailed 95% highest density interval (HDI) across the distribution of samples for each visit, a significant difference was defined as a HDI not spanning zero. The effect of ascent to high altitude and exercise at altitude were examined by looking at the interaction effect between the control and altitude group for the aforementioned paired time points.

Additional variables of the filter detection task, including perceptual sensitivity, perceptual bias, and metacognitive bias, are not fit hierarchically within the Hmeta-d model, so can be compared using standard frequentist statistics. Repeated measures analysis of variance (RANOVA) was used to compare these measures between visits with a 5% significance level. To compare the self-report scores between the altitude and control groups for each visit, responses were first tested for normality using an Anderson–Darling test, then if normal compared using a two-tailed *t*-test and if nonparametric compared using a Wilcoxon rank-sum test. Uncorrected *p*-values are presented and a Bonferroni correction for multiple comparisons between the two groups has been used to adjust the significance level, *p* = 0.05/20 = 0.0025.

To investigate the relationship between metacognitive ability and AMS, linear regression models were used to compare metacognitive performance from baseline (visit 1) and arrival at high altitude (visit 2) with AMS symptom burden over the trek. A significant relationship between the covariate and log of Mratio was defined as a two-tailed HDI that does not span zero across the beta samples for the covariate.

#### Examining baseline psychology

Hierarchical cluster analysis, similar to the method outlined by Abdallah et al. (2019), was used to assess the relationship between measures of symptoms of AMS on ascent to high altitude and baseline psychological state. Means of the daily measures of Lake Louise AMS score and oxygen saturations from the trek were included to represent AMS symptom burden. Only self-report scores that referred to individuals' “usual state” rather than the present moment or preceding week were included in the cluster analysis to best represent their baseline psychological state. Additionally, the Multidimensional Assessment of Interoceptive Awareness was excluded because it only strongly clustered with itself.

Subsequently, baseline psychological state was represented using State-Trait Anxiety Inventory—Trait only, Anxiety Sensitivity Index, Mobility Inventory for Agoraphobia, U.K. Biobank Neuroticism Scale, and Epworth Sleepiness Scale. These questionnaires were all completed during the baseline test at sea level in Birmingham. Before clustering, all variables were adjusted so that larger numbers represented a “worse” result and normalized through a z-transformation. The ability of the above variables to predict Lake Louise AMS score was further investigated using linear regression modeling.

## Results

### The effect of altitude on metacognition

There was no significant difference in Mratio between visit 1 (140 m) and visit 2 (2,624 m), HDI −0.2095, 0.6285. Additionally, there was no significant interaction effect (the effect of altitude) for the difference between visit 1 and 2 in the altitude group using control group as a comparison dataset (HDI 0.5525, −0.5699). Neither was there a significant difference in the additional filter detection task variables between the two visits: perceptual sensitivity (RANOVA, *F* = 0.5435, *p* = 0.4660), perceptual bias (RANOVA, *F* = 1.1436, *p* = 0.2924), and average confidence (RANOVA, *F* = 0.0001, *p* = 0.9918).

### The effect of a daily trek on metacognition

There was no significant difference in Mratio between visit 2 (2,624 m) and visit 3 (2,728 m, after an 11-day trek), HDI −0.5203, 0.4295. Additionally, there was no significant interaction effect (the effect of daily exercise) between visit 2 and 3 in the altitude group using the control group as a comparison dataset (HDI 1.6060, −0.1156). Neither was there a significant difference in the additional filter detection task variables between the two visits: perceptual sensitivity (RANOVA, *F* = 0.8250, *p* = 0.3701), perceptual bias (RANOVA, *F* = 0.3104, *p* = 0.5811), and average confidence (RANOVA, *F* = 1.9743, *p* = 0.1691).

### Metacognitive ability and AMS

The first regression model fitting Mratio, Lake Louise AMS score, and oxygen saturations from visit 2 (2,624 m) in the altitude group showed no significant association between metacognitive performance at that particular time point and corresponding Lake Louise AMS score (HDI −6.293, 0.2354) or oxygen saturations (HDI −0.4579, 0.3530). The second regression model fitting Mratio from visit 1 in the altitude group and average Lake Louise AMS score, oxygen saturations, and heart rate from the highest camp on the expedition (4,800 m) found no association between metacognitive performance at baseline and measures of symptom burden at the highest camp on the expedition: Lake Louise AMS score (HDI −0.1647, 0.5173), oxygen saturations (HDI −0.2021, 0.4682), and heart rate (HDI −0.3988, 0.1996).

### Questionnaires

The results of the self-report questionnaires from the baseline test of the control and altitude groups are shown in Table 1. There was no significant difference in baseline psychological measures or physical activity between the control and altitude groups, other than two subcomponents of the Multidimensional Assessment of Interoceptive Awareness questionnaire: noticing and not worrying. The daily measures of Lake Louise AMS score and cardiorespiratory function, recorded during a daily medical examination, are shown in Table 2. A total of 8/18 (44%) participants on the expedition developed AMS (defined as a Lake Louise AMS score ≥3), which was mild (Lake Louise AMS score <6) in all but 1 participant. This participant required premature evacuation from the highest camp due to concerns from the medical team.

**Table 1.** Self-Report Questionnaire Measures from Baseline Testing (in Birmingham, 140 m) for the Control and Altitude Group

Questionnaire	Control group, median ± interquartile range	Altitude group, median ± interquartile range	Normally distributed	*p*
MAIA—Noticing	3.6 ± 1.3	2.9 ± 1.3	False	0.1824
MAIA—Not distracting	3.0 ± 1.3	1.8 ± 1.3	False	<0.001^*^
MAIA—Not worrying	2.7 ± 1.0	3.8 ± 0.7	False	<0.001^*^
MAIA—Attention regulation	3.0 ± 0.7	2.7 ± 1.6	False	0.4955
MAIA—Emotional awareness	3.4 ± 1.4	3.0 ± 1.8	False	0.1196
MAIA—Self-regulation	3.0 ± 1.8	2.4 ± 2.0	False	0.3919
MAIA—Body listening	2.3 ± 1.3	2.2 ± 1.7	False	0.2020
MAIA—Trusting	4.0 ± 0.7	4.0 ± 1.0	False	0.5091
Fatigue Severity Scale	3.3 ± 1.2	2.9 ± 1.8	False	0.1208
Epworth Sleepiness Scale	7.5 ± 6.0	7.0 ± 3.0	False	0.6912
State-Trait Anxiety Inventory—State	33.5 ± 10.0	29.0 ± 14.0	False	0.2218
State-Trait Anxiety Inventory—Trait	38.0 ± 20.0	30.5 ± 8.0	False	0.0443
Center for Epidemiological Studies Depression Scale	11.5 ± 9.0	5.0 ± 6.0	True	0.0263
Positive and Negative Affect Scale—Positive	31.5 ± 10.0	39.0 ± 11.0	False	0.1130
Positive and Negative Affect Scale—Negative	14.5 ± 6.0	13.0 ± 6.0	False	0.4842
Mobility Inventory for Agoraphobia—Alone	1.4 ± 0.4	1.1 ± 0.1	False	0.0079
Mobility Inventory for Agoraphobia—Accompanied	1.2 ± 0.3	1.0 ± 0.1	True	0.0277
Anxiety Sensitivity Index	21.5 ± 14.0	10.5 ± 5.0	False	0.0028
U.K. Biobank Neuroticism Scale	2.5 ± 6.0	1.5 ± 4	True	0.2077
WHO Global Physical Activity Scale (metabolic equivalent minutes)	3900.0 ± 4580.0	3660.0 ± 3040.0	False	0.7879

An Anderson–Darling test was used to check whether data for each questionnaire were from a normal distribution reported as “True” for parametric data and “False” for nonparametric data; the two groups were then compared using a two-tailed *t*-test or Wilcoxon rank-sum test respectively.

Uncorrected *p*-values are presented. A Bonferonni correction for multiple comparisons was used to adjust the level of significance to 0.0025; significant values are marked with an asterix (^*^).

MAIA, Multidimensional Assessment of Interoceptive Awareness.

**Table 2.** Daily Physiological Measures from the High-Altitude Expedition (Mean ± Standard Deviation) and Prevalence of Acute Mountain Sickness (Lake Louise Acute Mountain Sickness Score ≥3) Recorded During the Daily Medical Review Each Morning

Day of expedition	Altitude, m	Oxygen saturations, %	Heart rate	Respiratory rate	Systolic blood pressure, mmHg	Diastolic blood pressure, mmHg	Daily AMS prevalence
1	1,650	94.9 ± 2.0	63.2 ± 9.5	14.7 ± 2.4	112.7 ± 8.2	69.1 ± 7.4	0
2	2,624	93.6 ± 2.0	61.1 ± 12.9	14.7 ± 1.9	111.8 ± 10.6	72.2 ± 7.9	0
3	2,624	91.3 ± 1.9	65.4 ± 12.5	15.2 ± 2.3	112.7 ± 10.7	72.9 ± 8.6	0
4	3,240	92.2 ± 1.7	62.1 ± 13.4	14.9 ± 2.4	113.2 ± 10.4	74.4 ± 5.3	0
5	3,240	89.2 ± 2.5	68.9 ± 16.2	16.3 ± 3.4	111.9 ± 9.4	70.4 ± 6.3	2
6	3,300	89.8 ± 2.5	67.7 ± 14.5	16.5 ± 3.1	116.8 ± 9.4	72.4 ± 6.3	0
7	3,850	88.6 ± 2.7	69.6 ± 13.7	15.4 ± 2.3	116.6 ± 9.2	74.3 ± 6.1	1
8	3,850	85.8 ± 3.4	65.9 ± 8.6	16.1 ± 2.6	117.4 ± 7.4	75.6 ± 6.2	4
9	4,800	87.4 ± 2.9	67.4 ± 13.8	17.8 ± 3.1	117.1 ± 9.7	75.1 ± 6.4	2
10	4,800	81.1 ± 4.5	71.8 ± 10.3	19.4 ± 3.5	120.4 ± 10.3	73.9 ± 6.6	5
11	4,800	80.5 ± 4.8	69.6 ± 11.6	18.6 ± 3.6	118.3 ± 10.7	73.1 ± 7.6	2
12	3,850	82.6 ± 3.1	66.8 ± 12.4	18.7 ± 4.1	130.1 ± 11.2	81.2 ± 8.8	1
13	3,240	88.9 ± 1.8	66.9 ± 14.9	17.1 ± 2.6	119.9 ± 12.9	75.9 ± 8.7	1
14	2,728	91.2 ± 2.4	66.1 ± 13.3	17.8 ± 2.7	116.7 ± 10.9	75.8 ± 7.1	1
15	2,728	91.6 ± 1.6	71.9 ± 16.4	17.7 ± 2.1	117.3 ± 10.5	73.2 ± 7.3	0
16	1,650	92.2 ± 1.2	67.2 ± 11.5	17.5 ± 2.5	113.3 ± 9.7	69.6 ± 9.2	0
17	1,650	94.6 ± 1.4	61.5 ± 14.2	16.2 ± 1.5	110.8 ± 9.5	69.1 ± 8.2	0

AMS, acute mountain sickness.

### Baseline psychology and AMS symptoms

**FIG. 3.**
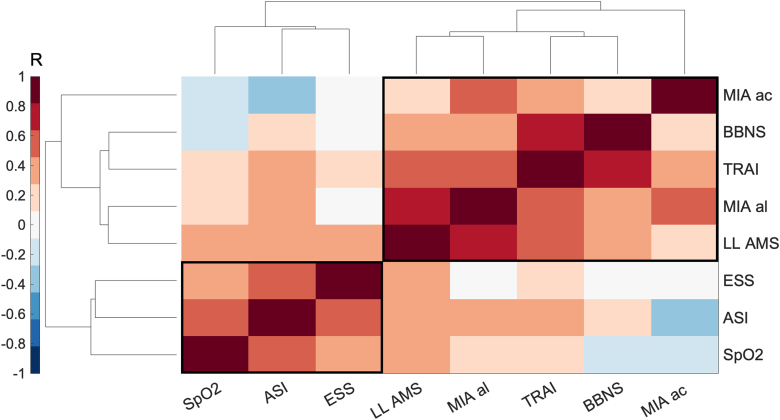
Clustergram: a correlation matrix of measures of AMS symptom burden over a high-altitude trek and baseline psychological measures recorded at sea level before the expedition. Symptom burden was represented by mean LL AMS score and mean oxygen saturations (SpO2) over the 11-day trek. Baseline psychology was represented by the following self-reported measures, TRAI, ASI, MIA al and MIA ac, BBNS, and ESS. Strength of correlation is measured as a Pearson's *R*-value displayed in the *color* bar. The relationship between the groups of measures is demonstrated by the height of the dendrogram branches and the distance between neighboring branches (in arbitrary units). AMS, acute mountain sickness; ASI, Anxiety Sensitivity Index; BBNS, U.K. Biobank Neuroticism Scale; ESS, Epworth Sleepiness Scale; LL AMS, Lake Louise Acute Mountain Sickness; MIA ac, Mobility Inventory for Agoraphobia—accompanied; MIA al, Mobility Inventory for Agoraphobia—alone; TRAI, State-Trait Anxiety Inventory—Trait only.

The results of the hierarchical cluster analysis are illustrated in Figure 3; the optimal number of clusters for this model was two. The first cluster was composed of mean oxygen saturation from the trek, Anxiety Sensitivity Index, and Epworth Sleepiness Scale. The second cluster included mean Lake Louise AMS score from the trek, Mobility Inventory for Agoraphobia—Alone and Accompanied, State-Trait Anxiety Inventory—Trait, and U.K. Biobank Neuroticism Scale.

To explore whether any of these measures were predictive of the Lake Louise AMS score, they were fitted into a linear regression model: number of observations 16, degrees of freedom 8, root mean squared error 0.4546, adjusted *R*^2^ 0.7926, *F*-statistic 9.1879, and *p* = 0.0028. The assumptions of linear regression, including normality of the residuals and homoscedasticity were met.

In this model, Epworth Sleepiness Scale (β_1_ = 0.5170, *p* = 0.0314) and Mobility Inventory for Agoraphobia—Alone (β_1_ = 1.1574, *p* = 0.0026) were predictive of Lake Louise AMS score. The other variables did not have a significant relationship with Lake Louise AMS score: mean oxygen saturation (β_1_ = −0.0082, *p* = 0.9750), State-Trait Anxiety Inventory—Trait (β_1_ = −0.0175, *p* = 0.9432), Anxiety Sensitivity Index (β_1_ = −0.3495, *p* = 0.1750), Mobility Inventory for Agoraphobia—Accompanied (β_1_ = −0.6083, *p* = 0.0675), and U.K. Biobank Neuroticism Scale (β_1_ = 0.0314, *p* = 0.9064).

## Discussion

AMS is a poorly understood condition, in which symptoms are inadequately explained by measures of peripheral physiology. Interoceptive performance, as measured by the filter detection task, was not impaired on ascent to high altitude and did not demonstrate any relationship with AMS symptoms. Average Lake Louise AMS score at high altitude clustered with self-reported measures of anxiety, agoraphobia, and neuroticism taken at sea level, but not average oxygen saturations at high altitude; this demonstrates the potential role individuals' psychology has in explaining their AMS symptom burden.

This finding supports previous work showing that baseline trait anxiety was a significant predictor of AMS on a high-altitude trek in the Himalayas (Boos et al., 2018). However, this is the first time the relative contributions of oxygen saturations and measures of anxiety (and other psychological traits) to AMS symptomology have been investigated. Interestingly, these findings have not been corroborated in hypoxic chamber studies at sea level (Niedermeier et al., 2017), which may reflect the different physiological responses to normobaric and hypobaric oxygen (Millet et al., 2012), or indeed the additional stresses of an expedition environment compared with a controlled laboratory environment.

There was no change in metacognitive performance between visit 1 at 140 m and visit 2 at 2,624 m; therefore, ascent to altitude did not act as an “interoceptive challenge.” This may reflect the low incidence of AMS in our expedition group of predominantly experienced mountaineers (Table 2); the mean group Lake Louise AMS score at visit 2 (2,624 m) was 0.83.

Neither was there a change in metacognitive performance between visit 2 and 3 after completion of an 11-day trek; therefore, daily exercise at altitude did not act as “interoceptive training.” Unfortunately, the trek was not as arduous as anticipated by the researchers. Additionally, the altitude group had a highly variable level of baseline activity level, with 74% of the group reporting >2,000 minutes of metabolic equivalent time per week according to the Global Physical Activity Questionnaire (World Health Organization, 2002). Thus, it is possible that the trek did not present an adequate training stimulus to alter metacognition.

Metacognitive performance on arrival at altitude (visit 2) was not related to Lake Louise AMS score or oxygen saturations at that time point, neither was baseline metacognitive performance (visit 1) predictive of AMS symptom burden (Lake Louise AMS score, oxygen saturations, or heart rate) at the highest point of the trek. It is possible that the filter detection task was not sufficiently sensitive to detect subtle changes in metacognition, particularly given the low prevalence of AMS on the expedition. Although, this same task has previously proven sensitive enough to detect interoceptive differences between asthma patient subgroups experiencing different levels of symptom severity (Harrison et al., 2021c).

Previous work has validated the filter detection task in participant groups with asthma and anxiety (Harrison et al., 2021b, 2021c), with interoceptive ability appearing to relate to symptom burden in these two conditions. However, this is the first study that has investigated the contribution of interoceptive ability to AMS and as such warrants larger validation studies. Ongoing work is taking place to optimize the filter detection task to make it more amenable to further field research in larger cohorts (Harrison et al., 2021a; Nikolova et al., 2021).

Mood and physical fatigue appear to be important perceptual modulators impacting individuals' symptom reporting (Finnegan et al., 2021; Harrison et al., 2021c), therefore, we investigated the impact of these factors on AMS symptom burden in our cohort with hierarchical cluster analysis. Average Lake Louise AMS score over the trek clustered with self-reported measures of anxiety, agoraphobia, and neuroticism taken at sea level. Agoraphobia is a type of anxiety disorder where individuals fear being in circumstances where escape may be difficult (American Psychiatric Association, 2013). It has previously been linked to dysfunctional interoceptive processes, where individuals have increased perceptual sensitivity paired with a propensity to misconstrue bodily sensations as dangerous, leading to panic (Breuniger et al., 2017).

Neuroticism has also been linked to interoceptive sensitivity (Pearson and Pfeifer, 2020) and certainly contributes to sensory perception in the Bayesian Brain, with neurotic individuals being predisposed to negative interpretations of sensations. Therefore, the clustering of Lake Louise AMS score with such psychological factors may represent a level of perceptual impairment in AMS prone individuals not detected by the filter detection task.

When fitted into a linear regression model, only two of the variables had a significant relationship with Lake Louise AMS score: Epworth Sleepiness Score and Mobility Inventory for Agoraphobia—Alone. Although a more specific symptom, sleepiness may be a manifestation of general fatigue, which is a theorized presentation of interoceptive dyshomeostasis (Stephan et al., 2016). However, this predictive relationship must be interpreted with caution given the sample size of this study and the number of variables included in the linear regression model. The authors suggest the limited inference that baseline psychological factors contributed to AMS symptom reporting in this study.

Notably, oxygen saturations did not cluster with Lake Louise AMS score and did not have a predictive relationship in the linear regression model. This suggests that individuals' psychological state may have contributed more to their experience of AMS symptoms on the trek than their oxygen saturations. Thus, although hypoxia is a well-established trigger of AMS development (Broessner et al., 2016), these findings suggest that psychological factors play an important role in modulating AMS symptom experience, which in turn is measured by the Lake Louise AMS score.

Indeed, Lake Louise AMS score is a general symptom score assessing headache, gastrointestinal symptoms, fatigue, dizziness, and ability to function with these symptoms. However, it is important to emphasize the impact of individuals' psychology on Lake Louise AMS scores given its widespread use by researchers and commercial trekking agencies alike.

We suggest that Lake Louise AMS scores should be interpreted with caution to prevent instances of serious pathology being missed. Furthermore, given the significant contribution of baseline psychological factors to subjective AMS symptoms, we suggest that questionnaires are unlikely to provide an objective measure of AMS, which is sorely needed to facilitate the prediction of its life-threatening complications, such as high-altitude cerebral edema and high-altitude pulmonary edema.

### Limitations

The study had a small sample size, with only 18 participants in each group. A larger sample size would allow for exploratory factor analysis and stratification of participants into behavioral phenotypes.

Furthermore, participation in high-altitude treks represents a rather niche interest that likely introduced some selection bias into this study. However, baseline questionnaires (shown in Table 1) demonstrated no significant difference in psychological measures or usual physical activity between the two groups.

We hypothesized that ascent to high altitude would impair interoceptive performance. However, the interoceptive tests took place at only moderate altitudes of 2,624 and 2,728 m, inducing low levels of AMS in our study group. This was largely for pragmatic reasons, to allow testing to take place in an indoor environment, as the trekking group camped at higher altitudes. The trek itself involved a high camp of 4,800 m though, which is comparable to many commercially available treks in the greater ranges.

## Conclusions

AMS remains a poorly understood condition, in which symptom burden is inadequately explained by measures of peripheral physiology. Interoceptive performance, as measured by the filter detection task, was not impaired in this study on ascent to altitude, or improved by daily exercise, and did not demonstrate any relationship with AMS symptoms. However, AMS symptoms were more closely related to self-reported psychological measures than oxygen saturations, demonstrating the contribution of psychological factors to individuals' experience of AMS symptoms. Therefore, we advise caution in the interpretation of Lake Louise AMS scores by researchers and commercial trekking companies alike to ensure serious cases of AMS are not missed.

## Supplementary Material

Supplemental data

## Preprint

An earlier draft of this article was posted as a preprint at bioRxiv (DOI: 10.1101/2021.07.22.451589v1).
