# Supplementary material for: Baseline Psychological Traits Contribute to Lake Louise Acute Mountain Sickness Score at High Altitude
Source: High Alt Med Biol. 2022 Mar 28;23(1):69–77. doi: 10.1089/ham.2021.0073 (PMC8982137; doi:10.1089/ham.2021.0073)
Supplement: Supplemental data [file Supp_Data.docx]

**Supplemental Information**

**Respiratory Filter Detection Task**

We developed the respiratory filter detection task used in this study (Harrison *et al.*, 2020) by adapting the inspiratory resistance task used by Garfinkel et al., (2016a), to measure respiratory interoceptive ability. To interpret the results of this interoceptive test, its psychometric properties must first be fully understood, including whether there are learning effects on repeated measurement. Additionally, the minimum number of trials required to accurately determine metacognitive ability needs to be assessed to minimize the duration of the test, making it more applicable to clinical practice. Therefore we tested the following two hypotheses in this study: (i) there would be no learning effect on repeated completion of the filter detection task, and (ii) forty trials of the filter detection task would be equivalent to sixty trials in its calculation of metacognitive ability.

***Learning Effect***

To investigate whether there was a learning effect on repeated measures of the filter detection task, we used data from the control group, who completed the test three times without any “interoceptive training” or “challenges” between tests. The HMeta-d model was fit for each pair of tests (visit 1 and 2, visit 2 and 3, and visit 1 and 3), whereby individual participant data from each test is drawn from a multinomial distribution and the dependence caused by repeated measures within subjects is accounted for. To determine the significance of group differences in Mratio estimates of each of the test pairs, the HDI was calculated across the distribution of sample differences from each of the time points, as previously described for the Hmeta-d model (Fleming, 2017). A two-tailed 95% HDI that does not span zero determines a significant difference between the datasets.

There was no significant difference in metacognitive performance (Mratio) between visit 1 and 2 (HDI, -0.2040, 1.0173), visit 2 and 3 (HDI, -0.8549, 0.7159), or visit 1 and 3 (HDI, -0.1718, 1.0186). Neither was there a significant difference in any of the additional filter detection task variables between the three visits: perceptual sensitivity (number of filters), RANOVA, F = 2.74, p = 0.303; perceptual bias (bias towards yes or no), RANOVA, F = 2.62, p = 0.314; and metacognitive bias (average confidence), RANOVA, F = 0.277, p = 0.962.

This is the first study to have carried out repeated measures of the filter detection task (Harrison *et al.*, 2020). There was no learning effect on repeated completion of the task, validating its use in longitudinal studies. The control group alone was used to study this hypothesis so as to avoid the potential confounders of ascent to high altitude and daily trekking that occurred in the altitude group.

***Forty Versus Sixty Trials of Filter Detection Task***

We hypothesized that 40 trials of the filter detection task would be equivalent to 60 trials in calculating an individual’s metacognitive sensitivity (Mratio). Again, this hypothesis was tested using the control group, as their results have fewer confounding factors, remaining independent from the effect of ascent to altitude and daily trekking. Similar to hypothesis 4, the HMeta-d model was fit separately to each timepoint, using two sets of data within a multinomial model: 1) all available trials, and 2) only the first 40 trials of each participant. Then a HDI was calculated across the distribution of sample differences from each of the data comparisons described in hypothesis 1.

There was no significant difference in metacognitive performance as estimated using 40 trials versus 60 trials at visit 1 (HDI -0.4580, 0.3612), visit 2 (HDI -0.7707, 0.6866), nor visit 3 (HDI -0.5045, 0.8718). Similar to 60 trials, there was no significant difference in the additional filter detection task variables between the three visits using 40 trials: perceptual sensitivity (RANOVA, F = 2.74, p = 0.303); perceptual bias (RANOVA, F = 0.976, p = 0.630); and average confidence (RANOVA, F = 0.318, p = 0.944).

The perceived value of using only 40 trials rather than 60 trials was to reduce testing time, and therefore make it more amenable for integration into clinical practice. The control group was used to compare trial numbers to avoid the confounding features of the expedition to high altitude. However, throughout the course of the study we found the time difference between 60 and 40 trials to be minimal, with explaining the task and establishing the threshold number of filters to be the time-consuming stages of each test. As the time-saving is minimal and we know from model simulations that increasing the number of trials increases our confidence in the HMeta-d model (Fleming, 2017; Harrison *et al.*, 2020), we recommend continuing to use 60 rather than 40 trials in the filter detection task.

**Study Participants**

The inclusion and exclusion criteria for participant recruitment are listed below.

Inclusion criteria

- All participants were willing and able to provide informed consent.
- All participant were adults aged 18 – 80 years.
- Only non-smokers were recruited.

Exclusion criteria

The participant did not enter the study if ANY of the following apply:

- Significant cardiac disease (e.g. heart failure, or pacemaker).
- Significant neurological disease (e.g. stroke, or neurodegenerative disease).
- Significant psychiatric disease (active treatment under psychiatric care).
- Significant metabolic disease (e.g. insulin dependent diabetes).
- Other respiratory diseases (e.g. sleep apnoea, chronic obstructive pulmonary disease, bronchiectasis, other restrictive lung disease, or severe asthma).
- Inadequate understanding of verbal and written information in English.
- Smoking history >20 pack years.
- History of prescription and non-prescription drug dependency (including alcoholism).
- Smokers.
- Travel above 1500 metres in the previous month.
- Travel across two or more time zones in the previous month.
- Travel across one time zone in the previous week.
- People who have worked night shifts in the previous month.

**References**

Fleming, S. (2017) ‘HMeta-d: hierarchical Bayesian estimation of metacognitive efficiency from confidence ratings’, *Neurosci. Conscious.*, 1, p. nix007.

Garfinkel, S. *et al.* (2016) ‘Interoceptive dimensions across caridac and respiratory axes’, *Phil Trans R Soc B*, 371, p. 20160014.

Harrison, O. *et al.* (2020) ‘The Filter Detection Task for measurement of breathing-related interoception and metacognition’, *bioRxiv*. doi: 10.1101/2020.06.29.176941.
